# A New Method to Infer Causal Phenotype Networks Using QTL and Phenotypic Information

**DOI:** 10.1371/journal.pone.0103997

**Published:** 2014-08-21

**Authors:** Huange Wang, Fred A. van Eeuwijk

**Affiliations:** 1 Biometris, Department of Plant Sciences, Wageningen University, Wageningen, The Netherlands; 2 Centre for BioSystems Genomics, Wageningen, The Netherlands; 3 Netherlands Metabolomics Centre, Leiden, The Netherlands; Pennsylvania State University, United States of America

## Abstract

In the context of genetics and breeding research on multiple phenotypic traits, reconstructing the directional or causal structure between phenotypic traits is a prerequisite for quantifying the effects of genetic interventions on the traits. Current approaches mainly exploit the genetic effects at quantitative trait loci (QTLs) to learn about causal relationships among phenotypic traits. A requirement for using these approaches is that at least one unique QTL has been identified for each trait studied. However, in practice, especially for molecular phenotypes such as metabolites, this prerequisite is often not met due to limited sample sizes, high noise levels and small QTL effects. Here, we present a novel heuristic search algorithm called the QTL+phenotype supervised orientation (QPSO) algorithm to infer causal directions for edges in undirected phenotype networks. The two main advantages of this algorithm are: first, it does not require QTLs for each and every trait; second, it takes into account associated phenotypic interactions in addition to detected QTLs when orienting undirected edges between traits. We evaluate and compare the performance of QPSO with another state-of-the-art approach, the QTL-directed dependency graph (QDG) algorithm. Simulation results show that our method has broader applicability and leads to more accurate overall orientations. We also illustrate our method with a real-life example involving 24 metabolites and a few major QTLs measured on an association panel of 93 tomato cultivars. Matlab source code implementing the proposed algorithm is freely available upon request.

## Introduction

In animal and plant breeding, selection of superior genotypes for further crossing is an important objective. To achieve this objective, identification of quantitative trait loci (QTLs) can be a first step in the development of a breeding strategy; alternatively nowadays, estimation of genomic breeding values can be considered to form another initial step. Whether a breeding strategy is based on QTLs or genomic breeding values, multi-trait approaches offer clear advantages over single-trait approaches [1], [2]. In multi-trait models, correlations, or associations, between traits have a symmetrical nature and are not supposed to convey information about causal relationships. Nonetheless, causal inference in correlated traits has been attracting growing research interest since it allows predicting effects of external interventions, where the effects of QTLs on phenotypic traits can be interpreted to represent a specific class of interventions [3], [4].

Causal inference in correlated traits, or equivalently, the construction of directed phenotype networks was so far mainly based upon logic that involves underlying QTLs [5]. For the simplest system with two traits (*T_1_*, *T_2_*) and one QTL (*Q*), Schadt *et al*. [6] and Li *et al*. [7] presented different implementations of triad analysis to determine whether the three entities are interconnected in, what they called, causal (*Q*→*T_1_*→*T_2_*), reactive (*Q*→*T_2_*→*T_1_*) or independent (*T_1_*←*Q*→*T_2_*) manner. Further research efforts concerned the investigation of multi-locus and multi-trait systems. Aten *et al*. [8] developed a network edge orienting (NEO) method and software to 1) perform genetic marker selection for each trait and 2) infer pairwise relationships between traits, using local-structure edge orienting (LEO) scores. Specifically, the LEO scores were calculated according to the likelihoods of local structural equation models (SEMs), which integrated two traits and the markers selected for each of them. Li *et al*. [9] introduced another systematic method to first infer genetic architecture of multiple traits and then iteratively assess and refine the path model by means of covariance-based SEM. Neto *et al*. [10] proposed a QTL-directed dependency graph (QDG) approach that requires a priori estimation of QTLs for the traits and executes the following two steps: 1) learn an undirected network from phenotypic data; 2) infer causal direction for every edge in the undirected phenotype network by conditioning on detected QTLs. In the QDG algorithm, QTL mapping is treated independently from the construction of phenotype network. In contrast, a QTL-driven phenotype network (QTLnet) method was introduced to jointly infer a directed phenotype network and the associated genetic architecture for a set of correlated traits [11]. An adaptive lasso (AL) based method was presented to infer a gene regulatory network from gene expression and expression quantitative trait loci (eQTLs) data [12]. In their simulation studies, Logsdon and Mezey [12] compared the performance of five algorithms, i.e. the PC algorithm [13], the NEO algorithm, the QDG algorithm, the QTLnet algorithm and the AL algorithm. The results indicated that in the setting of tens of traits and QTLs, the QDG and the AL algorithms exhibited comparable performance but consistently outperformed the other three methods. Logsdon and Mezey [12] also considered a couple of other algorithms including the one proposed by Li *et al*. [9], but they were deemed computationally expensive. Therefore, the QDG and the AL algorithms will be regarded as two state-of-the-art methods in this field.

In practice, it has become fashionable to map QTLs for phenotypes of interest via genome-wide scans, since genotyping has become cheaper and easier thanks to the advancement of genome sequencing technologies. Contrariwise, phenotyping, and especially metabolic profiling and sensory assessment, is still expensive and time-consuming [14]. Thus, for phenotypic traits such as metabolites and sensory attributes, it is hard to obtain large sample sizes that provide sufficient power for detecting small to medium sized QTLs. And it is often the case that, given high-dimensional phenotypic and genetic data (i.e. large numbers of traits and QTLs vs. small numbers of samples), significant QTLs cannot be identified for each and every trait [15], [16]. In such cases, both the QDG and AL algorithms become inapplicable as they require at least one unique QTL for each trait studied [10], [12].

To construct directed phenotype networks, especially when some traits come without QTLs, we present in this paper a QTL+phenotype supervised orientation (QPSO) algorithm. Compared with the benchmark QDG algorithm, our proposed method is likewise based on a priori determination of an undirected phenotype network and QTLs for the traits, where we recommend estimation of initial QTLs using multi-trait QTL mapping methods [1], [17], [18]. Our QPSO algorithm implements a heuristic search different from that of the QDG algorithm and investigates a more comprehensive local structure at each step. More specifically, the QPSO algorithm takes into account the related phenotypic interactions in addition to QTLs when orienting an undirected edge between two traits. As a result, it can orient multiple undirected edges simultaneously. The performance of the QPSO and the QDG algorithms is compared through a series of simulations. The results show that our method has broader applicability and produces more accurate overall orientations. To demonstrate the QPSO algorithm empirically, we use it in combination with the PC-skeleton [13] to build a partially directed network that sheds light on causal relationships between 24 metabolites in ripe fruits of a tomato association panel.

## Method

### Causal inference in two correlated traits

Assume *Y*_1_ and *Y*_2_ are two correlated traits connected by an undirected edge in a phenotype network. The causal direction of *Y*_1_–*Y*_2_ should follow one of two scenarios: *Y*_1_→*Y*_2_ or *Y*_1_←*Y*_2_. The two causal models are considered likelihood equivalent because p(*Y*_1_)p(*Y*_2_|*Y*_1_) = p(*Y*_1_,*Y*_2_) = p(*Y*_2_)p(*Y*_1_|*Y*_2_). Thus, it is impossible to distinguish between *Y*_1_→*Y*_2_ and *Y*_1_←*Y*_2_, i.e. to orient *Y*_1_–*Y*_2_, using a maximum-likelihood criterion.

**Figure 1:**
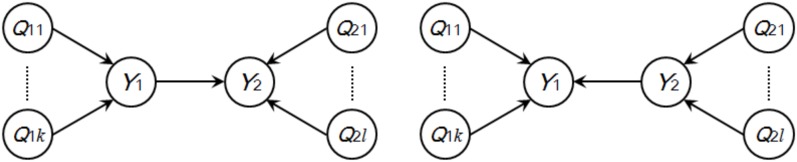
Candidate solutions to causal inference in two correlated traits. *Y*_1_ and *Y*_2_ are two traits correlated with each other; ***Q***_1_ = {*Q*_11_,…,*Q*_1*k*_} and ***Q***_2_ = {*Q*_21_,…,*Q*_2*l*_} denote QTLs for *Y*_1_ and *Y*_2_, respectively.

Neto *et al*. [10] presented a smart way to solve the problem of causal inference in two correlated traits. They introduced QTLs to *Y*_1_ and *Y*_2_ so as to get two expanded directed graphs as shown in Figure 1. The two expanded directed graphs are not likelihood equivalent since p(***Q***_1_)p(*Y*_1_|***Q***_1_)p(***Q***_2_)p(*Y*_2_|*Y*_1_,***Q***_2_)≠p(***Q***_2_)p(*Y*_2_|***Q***_2_)p(***Q***_1_)p(*Y*_1_|*Y*_2_,***Q***_1_), which can be further simplified as p(*Y*_1_|***Q***_1_)p(*Y*_2_|*Y*_1_,***Q***_2_)≠p(*Y*_2_|***Q***_2_)p(*Y*_1_|*Y*_2_,***Q***_1_). In this context, it is feasible to infer the causal direction of *Y*_1_–*Y*_2_ according to the maximum-likelihood criterion. More specifically, *Y*_1_–*Y*_2_ should be oriented in favor of the direction present in the model with higher likelihood, i.e. *Y*_1_→*Y*_2_ if p(*Y*_1_|***Q***_1_)p(*Y*_2_|*Y*_1_,***Q***_2_)>p(*Y*_2_|***Q***_2_)p(*Y*_1_|*Y*_2_,***Q***_1_) while *Y*_1_←*Y*_2_ if p(*Y*_1_|***Q***_1_)p(*Y*_2_|*Y*_1_,***Q***_2_)<p(*Y*_2_|***Q***_2_)p(*Y*_1_|*Y*_2_,***Q***_1_).

### Causal inference in local generalized phenotype networks

**Figure 2:**
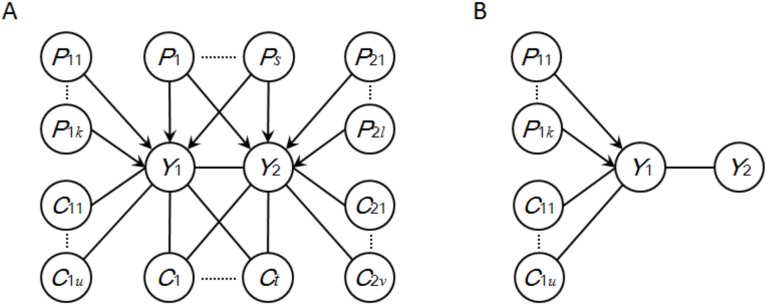
The general representations of resolvable LGPNs. *Y*_1_ and *Y*_2_ are two correlated traits; ***P***_1_ = {*P*_11_,…,*P*_1*k*_} and ***P***_2_ = {*P*_21_,…,*P*_2*l*_} are, respectively, the unique parent nodes of *Y*_1_ and *Y*_2_; ***P***_12_ = {*P*_1_,…,*P_s_*} are the common parent nodes of *Y*_1_ and *Y*_2_; ***C***_1_ = {*C*_11_,…,*C*_1*u*_} and ***C***_2_ = {*C*_21_,…,*C*_2*v*_} are the unique neighboring traits of *Y*_1_ and *Y*_2_; ***C***_12_ = {*C*_1_,…,*C_t_*} are the common neighboring traits of *Y*_1_ and *Y*_2_. Note that each of the neighboring traits of *Y*_1_ is nonadjacent to at least one of the parent nodes of *Y*_1_, and the same is true of *Y*_2_. Also note that ***P***_1_, ***P***_2_ and ***P***_12_ are allowed to have three different compositions: (1) a pure set of QTLs, if only genetic factors have been identified for *Y*_1_ and/or *Y*_2_; (2) a mixed set of QTLs and traits, if some traits in addition to QTLs have been determined to have causal effects on *Y*_1_ and/or *Y*_2_; (3) a pure set of traits, if only some traits have been found as causal factors of *Y*_1_ and/or *Y*_2_; in contrast, ***C***_1_, ***C***_2_ and ***C***_12_ only refer to those traits that are directly connected to *Y*_1_ and/or *Y*_2_ by an undirected edge. (A) The general representation of LGPNs where both *Y*_1_ and *Y*_2_ have parent nodes, and at least one of them has unique parent nodes; (B) the general representation of LGPNs where only *Y*_1_ has parent nodes.

In the context of Figure 1, *Y*_1_–*Y*_2_ is oriented by introducing parent nodes to *Y*_1_ and *Y*_2_, where the parent nodes are restricted to earlier identified QTLs. However, it is known that many molecular traits, such as metabolites and proteins, do interact with one another. This means that in addition to QTLs, some other traits may also have causal effects on *Y*_1_ and *Y*_2_. Therefore, these traits should also be included in the parent nodes of *Y*_1_ and *Y*_2_; or, at least, their potential effects on *Y*_1_ and *Y*_2_ should be taken into account when one is attempting to orient *Y*_1_–*Y*_2_. To make a comprehensive consideration of the local structure regarding *Y*_1_ and *Y*_2_, we present here the concept of local generalized phenotype network (LGPN) (Figure 2A), in which we include 1) QTLs identified for *Y*_1_ and *Y*_2_, 2) traits that have been determined as parent nodes of *Y*_1_ and *Y*_2_, 3) traits that are directly connected to *Y*_1_ and *Y*_2_ by undirected edges (these traits are hereinafter referred to as neighbouring traits of *Y*_1_ and *Y*_2_).

It has been demonstrated that the maximum-likelihood criterion can be employed to infer the direction of *Y*_1_–*Y*_2_ in the context of Figure 1. Inspired by this, we find a feasible solution to the problem of causal inference in LGPNs that meet the following two conditions: 1) both *Y*_1_ and *Y*_2_ have parents nodes and at least one of *Y*_1_ and *Y*_2_ has unique parent nodes; 2) each neighboring trait of *Y*_1_ is nonadjacent to at least one of the parent nodes of *Y*_1_, and the same is true of *Y*_2_. Assume in such a LGPN there are *n* undirected edges including *Y*_1_–*Y*_2_. As every undirected edge has two optional directions (i.e. either forward or backward), the total number of candidate directed graphs derived from that LGPN is then 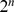. Verma and Pearl [19] have proved a theorem for the characterization of equivalent graphical models.

**Theorem:** Two directed acyclic graphs (DAGs) are likelihood equivalent if and only if they have the same skeletons and the same v-structures (A v-structure in a DAG G is an ordered triple of nodes (X, Y, Z) such that G contains the directed edges X→Y and Z→Y, and X and Z are not adjacent in G).

According to the theorem, we find that under the two aforementioned conditions, each of the 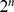 candidate directed graphs possesses a distinct set of v-structures (for detailed explanation please refer to File S1) and thus returns a distinct log-likelihood score 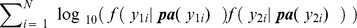, where *N* is the sample size, ***pa***(*X*) represents the parent nodes of trait *X*, and *f*() is a conditional probability density function with parameters replaced by the corresponding maximum-likelihood estimates. Accordingly, the locally optimal directed graph (LODG) among the 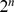 candidates should be the one with the highest log-likelihood score.

All undirected edges involving in a LGPN can be oriented simultaneously in the light of the corresponding LODG. These newly determined directed edges will then be employed to infer directions of some remaining undirected edges in the entire phenotype network. This leads to a heuristic search process, which will be described in detail in the following section. In the process of heuristic search, it might happen that some of the traits have never been assigned parent nodes in all of the previous steps. In cases where only *Y*_1_ or *Y*_2_, say *Y*_1_, has been determined with parent nodes, the maximum likelihood criterion is able to identify the LODG for a reduced LGPN (Figure 2B), and the log-likelihood score should be reformulated as 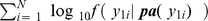. In particular cases where neither *Y*_1_ nor *Y*_2_ has unique parent nodes, the maximum likelihood criterion fails to infer direction of *Y*_1_–*Y*_2_. This means that the consideration of LGPN regarding *Y*_1_ and *Y*_2_ becomes a bit pointless and should be skipped.

In this study, we restrict ourselves to quantitative phenotypic traits and categorical QTL data, i.e., QTLs are represented by closest markers that can take one of two or three genotypes at that locus, depending on the type of population. Missing values in phenotypic and marker data are assumed to be estimated or imputed before that causal inference is applied. We also assume that a LGPN is a conditional linear Gaussian (CLG) model, in which discrete variables are not allowed to have continuous parents, and the joint distribution of continuous variables for every instantiation of discrete variables is multivariate Gaussian [20].

### Causal inference in an entire undirected phenotype network

**Figure 3:**
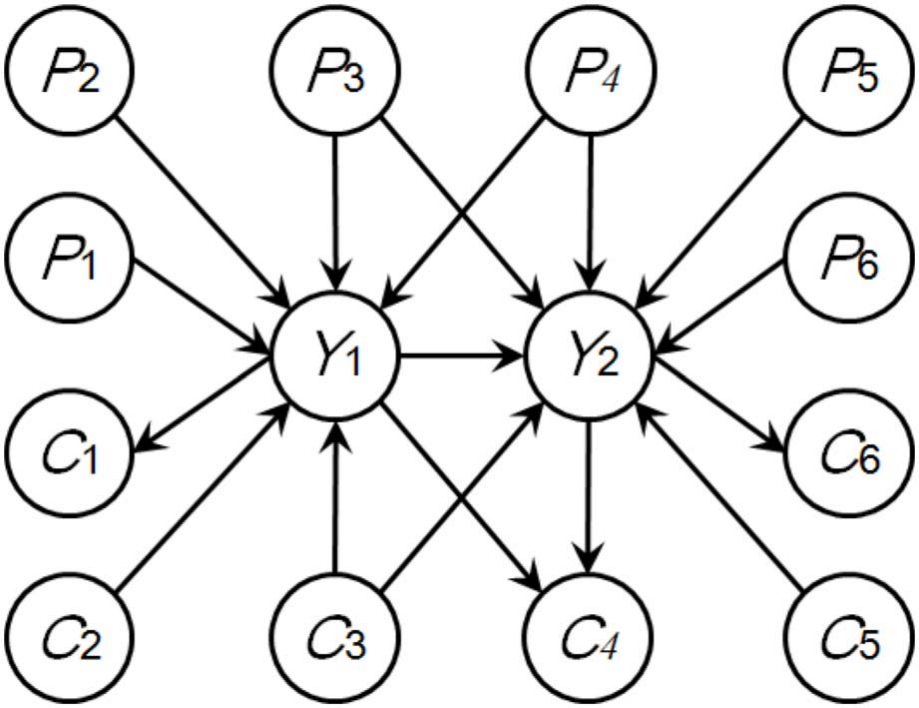
An example of LODG. *Y*_1_ and *Y*_2_ are two correlated traits; *C*_2_ and *C*_3_ are two traits that have been newly determined as parent nodes of *Y*_1_; *Y*_1_, *C*_3_ and *C*_5_ are three traits newly determined as parent nodes of *Y*_2_; *Y*_1_ is a newly determined parent node of traits *C*_1_ and *C*_4_; *Y*_2_ is a newly determined parent node of traits *C*_4_ and *C*_6_.

A LODG may introduce new parent nodes to some of the traits. As illustrated in Figure 3, *Y*_1_ is the newly determined parent node of *C*_1_ and *C*_4_. This updated causal information might subsequently enable or improve the orientation of the remaining undirected edges connecting to *C*_1_ and *C*_4_. Therefore, iterative implementation of causal inference in sequential LGPNs can finally orient as many edges as possible in an undirected phenotype network. This is, however, a typical heuristic search technique that has to be rerun from different starting points a number of times to avoid getting stuck in local optima. To this end, we exploit the Bayesian information criterion (BIC) score as a global evaluation metric to find the most likely fully or partially directed phenotype network obtained in multiple runs. The BIC score is a well-known penalized likelihood criterion that is often used to prevent overfitting the training data. It is formally defined as 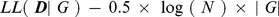, where ***D*** is the training data, *G* is the learnt network, 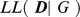 is the maximum log-likelihood, *N* is the sample size, and 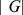 denotes the dimension of *G* [21].

In summary, our QPSO algorithm executes the following steps to perform causal inference in an entire undirected phenotype network, where we assume that the QTLs have been identified earlier by a multi-trait QTL mapping method like the ones described in [17] and [18].

1. Randomly choose a pair of traits that simultaneously satisfy two conditions: first, they are connected by an undirected edge; second, both of them have parent nodes and at least one of them has unique parent nodes.
2. Extract the LGPN (as illustrated in Figure 2A) with respect to these two traits.
3. Identify the LODG from all candidate directed graphs derived from that LGPN; update the phenotype network (i.e. orient all the corresponding undirected edges) according to the LODG.
4. Repeat steps (1), (2) and (3) until no more traits satisfying the two conditions mentioned in step (1) remain.
5. If the resulting phenotype network is partially directed, randomly choose a pair of traits that simultaneously satisfies two conditions: first, the traits are connected by an undirected edge; second, only one of them has parent nodes.
6. Extract the LGPN (as illustrated in Figure 2B) with respect to these two traits.
7. Identify the LODG from all candidate directed graphs derived from that LGPN; update the phenotype network according to the LODG.
8. Repeat steps (5) (6) and (7) until no more undirected edges can be oriented; store the overall orientation of the entire phenotype network.
9. Repeat steps (1) through (8) a number of times (this number is hereinafter referred to as the number of iterations); use the BIC score to evaluate each overall orientation and return the one with the highest score.

An implementation of the QPSO algorithm has been realized in Matlab. Thereinto, the probability density function of the CLG distribution and the BIC score are computed by calling functions in Bayes Net Toolbox (https://code.google.com/p/bnt/). Matlab source code is available from the authors upon request.

## Results

### Synthetic phenotypic and QTL data

**Figure 4:**
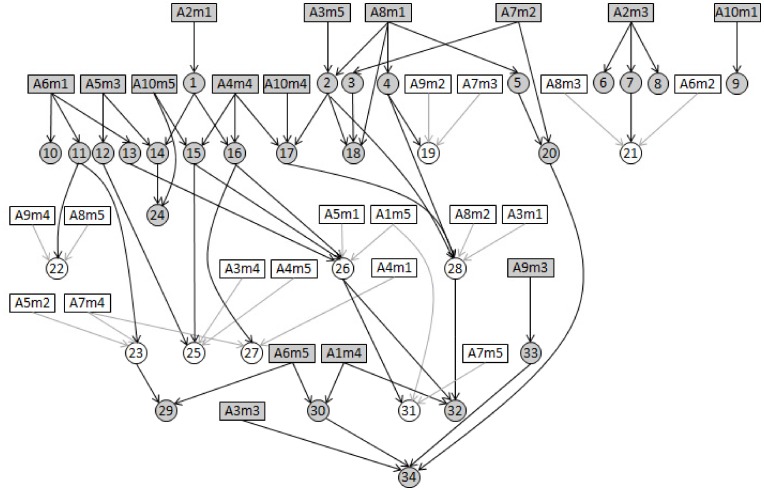
A synthetic QTL-phenotype network. This network consists of 31 QTLs, 34 traits and 74 directed edges. Traits are ordered by numerical numbers and QTLs are labelled in the form of ‘C*i*m*j*’ indicating the *j*-th marker on the *i*-th chromosome. Because only a part of QTLs were used in a third set of simulations, the nodes are further classified as follows: shaded rectangular nodes–QTLs present in the third set of simulations; clear rectangular nodes–QTLs absent in the third set of simulations; shaded circular nodes–traits provided with QTLs in the third set of simulations; clear circular nodes–traits provided without QTLs in the third set of simulations.

We followed the same protocol used in [10] to generate synthetic data for a simulation study creating phenotypic and marker data for an F2 population. A directed network composed of 65 nodes and 74 edges (Figure 4) was created by the *randomDAG* function in the R package ‘*pcalg*’ (http://cran.r-project.org/web/packages/pcalg/index.html). In this network, 34 nodes denoted phenotypic traits while the other 31 nodes represented QTLs. QTLs were randomly selected among 50 markers, with 5 markers unevenly distributed on each of 10 chromosomes. Observations of a trait were generated on the basis of linear regression model 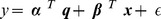, where ***q*** is a vector of marker scores (QTLs), ***x*** is a vector of traits, 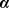 and 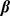 are the regression coefficients corresponding to ***q*** and ***x***, and 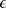 is the residual. To simplify exposition, we assumed quantitative traits and categorical QTL data, and allowed only additive genetic effects with an increment of 0.1 per allele. Specifically, QTL genotypes *aa*, *Aa* and *AA* were respectively encoded as 1, 2 and 3; the regression coefficient for genotype *aa* was uniformly drawn from [0.2, 0.4]; the coefficients for genotypes *Aa* and *AA* were then given by adding 0.1 and 0.2, respectively. Besides, the regression coefficient of a phenotype on one another was chosen uniformly from [0.5, 1], and the standard deviation of 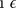 was randomly drawn from [0.1, 0.5]. A set of synthetic phenotype and QTL data is given in File S2.

### Simulation results

Our QPSO method is applicable to pre-learnt undirected or partially directed phenotype networks. There are a number of ways to learn undirected graphical models from data, including marginal and partial correlation analyses, as well as conditional independence tests. We consider the QDG algorithm still to represent a benchmark algorithm with which to compare our QPSO approach. The QDG algorithm uses an undirected phenotype network as reconstructed by the PC-skeleton algorithm as the starting configuration for edge orientation. For the comparative simulations, we also took the PC-skeleton as the method to arrive at an undirected phenotype network.

In a first set of 20 simulation runs, we evaluated the performance of the PC-skeleton algorithm using two indicators, recall and precision. Each simulation run was based on a distinct phenotypic dataset. Recall, also called true positive rate or sensitivity, measures the proportion of true edges that are retrieved in relation to the full set of true edges. Precision, or positive predictive value, measures the proportion of true (positive) edges in the set of identified edges (true and false positives). The higher recall and precision, the better the reconstruction of the network is. The results of our first set of simulations are shown in Table 1, where means and standard deviations for recall and precision are given. With increasing sample size, both recall and precision improved with respect to their means across simulation runs, while their standard deviations remained at a low level. In particular, given that in practice 100 individuals is a representative sample size for biological data like metabolites, a recall of 0.86 and a precision of 0.97 on average, is very encouraging. High mean value and low standard deviation indicate that the PC-skeleton algorithm can accurately and consistently recover an undirected network, using a reasonable sample size.

**Table 1:** Performance of the PC-skeleton algorithm in reconstructing the synthetic phenotype network across a series of 20 simulations.

Samplesize	Recall		Precision	
	mean	sd	mean	sd
100	0.86	0.06	0.97	0.03
200	0.94	0.03	0.97	0.03
300	0.96	0.03	0.98	0.03
400	0.98	0.03	0.98	0.03
500	0.99	0.03	0.98	0.02

The significance level of conditional independent tests used in the PC-skeleton algorithm was set at 0.01.

Given an undirected phenotype network pre-learnt by the PC-skeleton algorithm, our next step was to infer causal directions for edges in the network by exploiting associated QTLs. Both the QDG and QPSO algorithms are applicable to this problem when at least one QTL has been identified for each and every trait. In a second set of simulations we then made a comparative evaluation of the two edge orientation algorithms using the full set of QTL data and the earlier reconstructed undirected phenotype network. Results are presented in Table 2, where we give mean and standard deviation of the proportion of true positive edges that were correctly oriented for QDG using all QTLs and QPSO using all QTLs over 20 independent simulation runs. To achieve consistent results (i.e. small standard deviations) from multiple runs, the QDG algorithm claimed 1000 iterations [10] while our QPSO method required only about 10 iterations for each individual run. Two conclusions regarding the effectiveness of the two algorithms can be drawn from the comparative study. First, along with the increase of samples, the overall orientations obtained by both methods became increasingly accurate and consistent. Second, given the same sample size, the QPSO algorithm produced more accurate overall orientation than the QDG method, since the former always possessed a higher mean proportion of correctly oriented true edges combined with a comparable or slightly lower standard deviation.

**Table 2:** Comparative evaluation of three algorithms in overall orientation of the synthetic phenotype network.

Samplesize	QDG (using		QPSO (using		QPSO (using		PC (no use of	
	full QTLs)		full QTLs)		partial QTLs)		QTLs)	
	mean	sd	mean	sd	mean	sd	mean	sd
100	0.72	0.11	0.78	0.07	0.77	0.09	0.49	0.11
200	0.74	0.08	0.81	0.07	0.80	0.08	0.57	0.06
300	0.75	0.06	0.81	0.06	0.81	0.07	0.60	0.05
400	0.77	0.05	0.82	0.06	0.82	0.06	0.60	0.05
500	0.78	0.05	0.84	0.05	0.83	0.06	0.60	0.05

Sample size, means and standard deviations of the proportion of true positive edges that were correctly oriented across a series of 20 simulations.

The major advantage of the QPSO algorithm lies in the ability of inferring causal relationships between correlated traits when some or more of the traits do not have QTLs. To demonstrate this, in a third set of simulations, we blanked out a number of detected QTLs and then investigated the performance of the QPSO algorithm. We assumed that QTLs corresponding to the clear rectangular nodes in Figure 4 were not available for the reconstruction of the directed phenotype network, i.e., these QTLs were removed from the input of the QPSO algorithm. Results of this particular simulation study are summarized in the columns of Table 2 that are labelled QPSO using partial QTLs. It is obvious that QPSO still has reasonably good edge orientation even when a substantial proportion of the traits come without QTLs. Table 2 learns that given the same sample size, the overall orientation obtained by the QPSO algorithm with partial QTLs is getting refined when sample size increases, and is slightly inferior to the one obtained by the same algorithm with full QTLs, but nevertheless superior to the one obtained by the QDG algorithm with full QTLs.

To demonstrate the robustness of the QPSO algorithm, we elaborated on the results of the third set of simulations reported above. We selected five edges from the simulated phenotype network that differed with respect to the configuration of parent nodes for two correlated traits: (1) between traits 2 and 18, with the two traits having one common QTL (C8m1) and trait 2 having a unique QTL (C3m5); (2) between traits 1 and 16, with each trait having a unique QTL (C2m1 for trait 1 and C4m4 for trait 16); (3) between traits 16 and 26, with trait 16 having a unique QTL (C4m4) and trait 26 having no QTL; (4) between traits 13 and 26, with trait 13 having a unique QTL (C6m1) and trait 26 having no QTL; (5) between traits 26 and 31, with neither trait having QTL. We investigated the accuracy of orientations obtained by the QPSO algorithm for the five edges (Table 3). When sample size increased from 100 to 500, the two edges 2–18 and 13–26 were almost 100% correctly oriented, the average percentages of correct orientations improved from 65 to 95% for the edge 1–16, from 25 to 70% for the edge 16–26 and from 35 to 100% for the edge 26–31. The declining performance of our method on edge 16–26 than 1–16 was mainly due to error propagation in orientations. If an incorrect direction has been assigned to edge 1–16 in a previous step, it will affect the accuracy of orientation regarding edge 16–26. Likewise, an incorrect direction inferred for edge 16–26 will subsequently harm the orientation of edge 26–31. However, results in Table 3 indicate that our QPSO algorithm possesses higher accuracy in orientation of edge 26–31 than of 16–26. This is because the algorithm makes a full consideration on the neighborhood of trait 26 (i.e. the interactions between traits 13, 15, 16 and 26 were all taken into account) when orienting the edge 26–31, so that the negative impact of incorrect orientation of edge 16–26 can be counterbalanced, to some extent, by the positive effect of correct orientation of edge 13–26.

**Table 3:** Demonstration of the robustness of the QPSO algorithm.

Samplesize	Proportions of correct orientations of five edges				
	2→18	1→16	16→26	13→26	26→31
100	1.00	0.75	0.45	1.00	0.45
200	1.00	0.75	0.50	1.00	0.80
300	1.00	0.80	0.60	1.00	0.95
400	1.00	0.85	0.65	1.00	1.00
500	1.00	0.95	0.70	1.00	1.00

Proportion of correct edge orientation across 20 simulations for edges with varying parent configurations. Node numbers refer to Figure 4. Decimal numbers were the average values deduced from 20 independent runs in the third set of simulations.

Each run of the QPSO algorithm selects the best model according to the maximum-likelihood criterion. Nonetheless, in many cases, several models may have very close likelihoods, meaning that they are all compatible with the data. Therefore, it is critical to check the consistency of those competing models. Also based on the third set of simulations, we compared the best two models obtained by a single run of the QPSO algorithm for different sample sizes. The results (Table 4) show that for a given sample size, the best two models indeed possess very close BIC scores; but, more importantly, they are substantially the same, except for a handful of edges that are assigned with opposite directions in the two models. In view of the high consistency that exists between the best two models, we believe it will suffice to return only the best model as final output. All simulations were implemented in a 32 bit Intel(R) Core(TM) i5-2410 M 2.30 GHz 4 GB RAM machine. The computing time of a single run of the QPSO algorithm for each sample size studied is also included in Table 4.

**Table 4:** Comparison between the best two models obtained by a single run of the QPSO algorithm.

Samplesize	The best model			The second-best model			Computingtime (h)
	BIC score	different edges		BIC score	different edges		
100	−6.8990e+03	14→24 (√)		−6.9057e+03	14←24 (×)		0.89
200	−1.3308e+04	1→14 (√)	1←16 (×)	−1.3325e+04	1←14 (×)	1→16 (√)	1.27
		16→27 (√)	14←24 (×)		16←27 (×)	14→24 (√)	
300	−1.9663e+04	11→23 (√)	1←16 (×)	−1.9693e+04	11←23 (×)	1→16 (√)	2.18
		16→26 (√)	11←22 (×)		16←26 (×)	11→22 (√)	
			14←24 (×)			14→24 (√)	
400	−2.5944e+04	11→23 (√)	11←22 (×)	−2.5988e+04	11←23 (×)	11→22 (√)	2.76
		15→25 (√)	15←26 (×)		15←25 (×)	15→26 (√)	
500	−3.2333e+04	1→14 (√)	4←28 (×)	−3.2459e+04	1←14 (×)	4→28 (√)	3.30
		4→19 (√)	11←22 (×)		4←19 (×)	11→22 (√)	
		11→23 (√)			11←23 (×)		
		17→28 (√)			28←17 (×)		

Computing time was measured on a 32 bit Intel(R) Core(TM) i5-2410M CPU 2.30GHz machine with 4GB RAM. “different edges” were assigned with opposite directions in the best two models. (√) means the direction of that edge was inferred correctly, whereas (×) applies to the opposite case.

As explained in the Method section, the QPSO method returns fully or partially directed phenotype networks depending on the number of available QTLs. The PC algorithm, which is a further extension of the PC-skeleton algorithm, also returns partially directed phenotype networks but without using QTLs. To demonstrate the advantage of our QPSO method over the PC algorithm, in a final set of simulations we assessed the performance of the PC algorithm in the reconstruction of the simulated phenotype network. Results are shown in the last two columns of Table 2. The last four columns of Table 2 support the conclusion that for a given sample size and undirected phenotype network, the QPSO algorithm with partial QTLs orients correctly far more edges than the PC algorithm, which orients edges without using QTL information.

### Metabolic and QTL data collected in ripe tomato fruits

**Figure 5:**
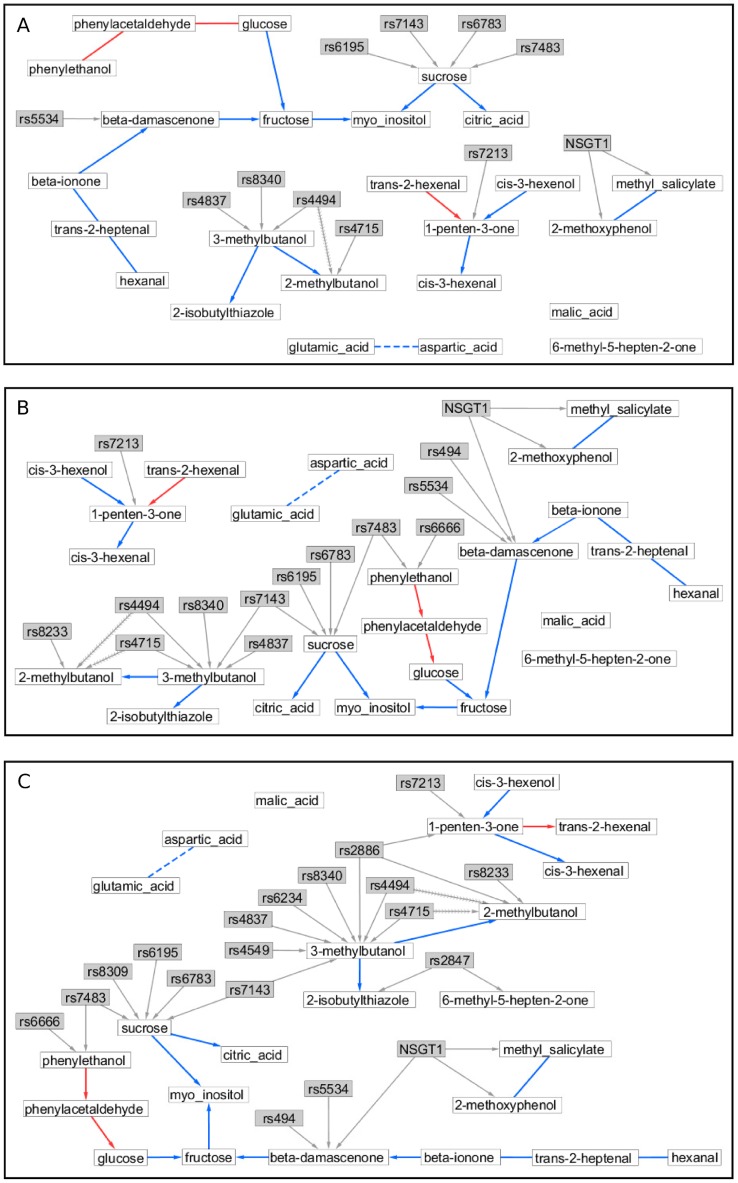
Three partially directed graphs describing the relationships among 24 metabolites in ripe tomato fruits. Clear nodes represent metabolites; shaded nodes denote QTLs identified for the metabolites. QTLs in (A), (B) and (C) were selected on the basis of –log_10_ (p*-*value) thresholds 5.5, 5.0 and 4.5, respectively. Grey edges link QTLs to the corresponding metabolites. Blue and red edges, without regard to their directions, were learnt by the PC-skeleton algorithm; their directions, if any, were inferred by the QPSO algorithm. Blue edges occur consistently throughout the three graphs representing different test levels for QTLs, while red edges do not. Solid and dashed edges indicate positive and negative correlations, respectively; fishbone edges are removed by post hoc causal reasoning.

Metabolic data were collected from ripe fruits of 93 tomato cultivars, an association panel provided by five breeding companies involved in the Centre for BioSystems Genomics tomato quality program (http://www.cbsg.nl/tomato.aspx). According to morphological characteristics of ripe tomato fruits, the 93 cultivars were categorized into three groups, labelled as beef, cherry and round. The three groups made up approximately 25%, 25% and 50% of the total collection. Metabolic profiling of cultivars was based on pooled fruit samples, where the sample for each beef or round cultivar mixed 12 fruits while the sample for each cherry cultivar contained 18 fruits. Sugars and acids were measured using the technique described in [22]. Volatiles were quantified using the method presented in [23]. In this study, we investigated a subset of 24 metabolites of special interest. The same set of metabolic data was studied in [24], where a detailed description of the measurements and the data can be found. Most of the metabolites strongly discriminated between cherry and non-cherry (i.e. beef and round) tomatoes, as was found by both principal component analysis and discriminant analysis [24]. Application of the PC-skeleton algorithm to reconstruct a phenotypic network between the 24 metabolites led to a network with 17 edges (Figure 5). The reconstruction was done choosing a rather strict test level of 0.01 for the conditional independence tests to arrive at a sparse but high confidence phenotypic network.

To find a list of QTLs driving the variation in the 24 metabolites, association analysis was performed using 600 SNPs in a multi-trait mixed model association mapping procedure that allowed for trait specific effects of pleiotropic QTLs. In addition, this mixed model contained intercept terms for the cherry and non-cherry groups to correct for this obvious type of population structure. To investigate the susceptibility of the QPSO algorithm to the amount of QTL information for orienting edges between metabolites, we selected QTLs at three levels significance. The more liberal the threshold, the greater the number of selected QTLs is. We adopted three closely together thresholds for the significance of the test for a QTL with an effect on any of the 24 metabolites at a given marker locus, corresponding to –log_10_ (p-value) = 4.5, 5.0, 5.5. At the strictest level of –log_10_ (p*-*value) >5.5, 11 QTLs were identified for seven metabolites (Figure 5A), with two QTLs that had pleiotropic effect on two metabolites. Of the 24 metabolites, 17 remained without QTL. Lowering the –log_10_ (p*-*value) for QTL detection to 5.0 led to four additional QTLs and more QTLs with pleiotropic effects: eight metabolites came with one or more QTLs, 16 stayed without QTLs (Figure 5B). At a –log_10_ (p-value) threshold of 4.5, a total of 19 QTLs were detected for 10 metabolites (Figure 5C). The metabolic and QTL data for this study are available in the File S3.

### Causal relationships among tomato metabolites

The QPSO algorithm was used to orient undirected edges between the metabolites. The results corresponding to QTLs selected at the three thresholds of –log_10_ (p*-*value) = 4.5, 5.0 and 5.5 are shown in Figures 5A, B and C, respectively. Comparison of the three graphs indicates that when more QTLs with relatively small effects enter the model, more traits tend to be associated with at least one QTL, and accordingly more undirected edges between traits can be oriented. The 11 QTLs for the seven metabolites in Figure 5A allowed 11 of the 17 edges to be oriented. For the 15 QTLs and 8 metabolites in Figure 5B and the 19 QTLs and 10 metabolites in Figure 5C, 13 edges out of the 17 could be oriented.

Among the 17 undirected edges between metabolites, 11 were oriented throughout the three graphs. We examined the consistency of the inferred directions of the 11 edges and found that only the edge connecting 1-penten-3-one and trans-2-hexenal came varied in direction across the test levels for QTLs. The directions of the other 10 edges were invariant to the changes in the amount of QTL information. This invariance of edge orientation provides a modest demonstration of the robustness of the QPSO algorithm.

**Figure 6:**
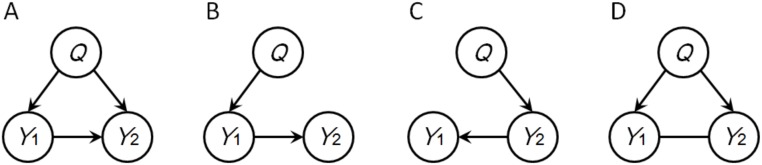
Test models in triad analysis. (A) a QTL *Q* has pleiotropic effects on two traits *Y*_1_ and *Y*_2_, *Y*_1_ is also a causal factor of *Y*_2_; (B) *Q* is identified for *Y*_1_, *Y*_1_ has a causal effect on *Y*_2_; (C) *Q* is identified for *Y*_2_, *Y*_2_ has a causal effect on *Y*_1_; (D) *Q* is identified for both *Y*_1_ and *Y*_2_, but the causal relationship between *Y*_1_ and *Y*_2_ is unclear.

After reconstruction of the directed network, an investigation of pleiotropic QTLs is possible in a post hoc analysis of the network. For example, in Figure 5C, initially the two QTLs rs4494 and rs4715 were pleiotropic for 3-methylbutanol and 2-methylbutanol. Simultaneously, 3-methylbutanol was identified to be a direct upstream metabolite of 2-methylbutanol. Did the two QTLs have pleiotropic effects on both traits, or, were their effects on 2-methylbutanol mediated via 3-methylbutanol? To answer this question, we used the BIC scoring metric to evaluate and compare the two models shown in Figure 6A and B, where *Q* denotes rs4494 or rs4715, *Y*_1_ and *Y*_2_ represent respectively 3-methylbutanol and 2-methylbutanol. It turned out that with respect to either of the two QTLs, the simplified model in Figure 6B possessed a higher BIC score, thereby providing a better fit to the observed data. Thus, we deleted from Figure 5C the two edges pointing from rs4494 and rs4715 to 2-methylbutanol. The same concern can be raised with respect to the QTL effect of NSG1 on methyl salicylate and 2-methoxyphenol. In this case, we failed to infer the causal relationship between the two metabolites due to lack of unique QTL. To this type of specific problems, Neto *et al*. [10] suggested a possible solution by comparing the likelihoods of the three models shown in Figure 6B, C and D. Here, we exploited the BIC score again and let *Q*, *Y*_1_ and *Y*_2_ denote NSG1, methyl salicylate and 2-methylbutanol, respectively. Comparative results indicated that the data best supported the pleiotropic model in Figure 6D, therefore the local structure of NSG1, methyl salicylate and 2-methylbutanol in Figure 5C should remain the same. The investigation to the reality of observed pleiotropic relations as described for Figure 5C was equally applied to Figures 5A and B.

Given the structure of the network, we estimated effects of traits on one another and of QTLs on traits. To that end, we regressed metabolites on QTLs and adjacent upstream metabolites. We discriminated between positive and negative associations among the metabolites according to the signs of fitted regression coefficients. The signs of QTL effects were not considered as they are somewhat arbitrary in the context of association mapping and binary markers such as SNPs.

The above directed network can be compared with undirected networks constructed on the basis of marginal and partial correlations, like a correlation network and a graphical Gaussian model (GGM), see Figure 5 and 9 as presented in [24]. Both these graphs look very dense despite the fact that only strongly significant correlations were displayed (*q*<0.05, as a false discovery rate procedure was chosen). From a dense graph with many variables incorporated, it is hard to arrive at meaningful interpretations. Compared with the results reported in [24], our findings obtained by the PC-skeleton algorithm in combination with the QPSO algorithm comprised a much sparser graph, with the additional advantages of showing (partial) directedness between traits and the influence of QTLs on traits. It should be remarked that between the three graphs, a central backbone coincided.

Although we reconstructed a directed network on a set of metabolites, the resulting network cannot be interpreted as an approximation to a metabolic network, a major reason being the absence of time course data. The metabolic data we analysed represented mean metabolite abundances obtained from grinding a number of fruits for a set of tomato genotypes. To get insight in biological pathways, we should measure series of chemical reactions occurring over relative short time frames within a cell, but the measurement and analysis of such time series still presents large challenges [25]. The value of a directed network like that of Figure 5 is that it allows to correctly quantifying the effects of QTL allele substitutions, say genetic interventions or perturbations, at a number of phenotypic traits simultaneously. For instance, changes at locus rs7213 will have an effect on the concentration of 1-penten-3-one, which will subsequently affect the concentration of cis-3-hexenal. In contrast, variations in the concentration of 1-penten-3-one will not influence the level of trans-2-hexenal, as trans-2-hexenal is an upstream metabolite of 1-penten-3-one. Another representative example is that if we attempt to control the concentration of 2-methylbutanol, we should be cautious about the allelic composition at loci rs4715, rs8396, rs8340, rs7143 and rs8233, since any genetic perturbation leading to an alteration in the concentration of 3-methylbutanol will then change the concentration of 2-methylbutanol.

From a biological point of view, Figures 5A, B and C present several interesting clusters. It is noteworthy that the major carbohydrates glucose and fructose are linked to sucrose and citric acid via myo-inositol. Whilst myo-inositol is synthesized from glucose, the recovery of the indirect link is remarkable, also considering that myo-inositol is linked to sucrose which can be broken down into glucose and fructose or alternatively into UDP-glucose and fructose. Another remarkable link is the one between beta-damascenone and beta-ionone both of which are break-down products of carotenoids [26]. Interestingly 6-methyl-5-hepten-2-one was not linked to these, despite being a carotenoid class volatile. This indicates that the latter open chained form likely stems from lycopene [27], potentially explaining why it is not linked to any of the former two metabolites. Furthermore, the negative correlation between aspartic acid and glutamic acid might be explained by the action of aspartate aminotransferase converting glutamate oxaloacetate to 2-oxoglutarate and aspartate. It is clear that the C5 and C6 volatiles were grouped together. Whilst intriguing that these are likely produced from the same precursors via lipoxygenases [28], one would speculate that the C5 and C6 volatiles should probably be disconnected, making the 1-penten-3-one (C5) mini hub linked to many C6 volatiles worth further investigation. Incidentally, both C5 and C6 volatiles were also found in different clusters previously [29]. Regarding the metabolites 3- and 2-methylbutanol, they both are likely leucine/isoleucine derived compounds and they were found linked to 2-isobutylthiazole before [29].

In summary, our partially directed network for the 24 tomato metabolites is clearly more concise and informative than those of conventional marginal and partial correlation analyses and allowed discriminating between direct and indirect metabolic responses to particular genetic perturbations in tomatoes. Following Valente *et al*. [4], it is exactly this type of information that is needed for predicting the effects of genetic interventions on sets of correlated phenotypic traits.

## Discussion

The QPSO algorithm is applied to pre-learnt undirected or partially directed phenotype networks. Correlation networks and GGMs are the most common models used to learn undirected graphs from biological data [24], [30], [31]. Bayesian networks (BNs) are considered a promising tool to recover partially directed biological networks [32]–[34]. Formally, a BN is a DAG that represents probabilistic conditional independence structures for a set of interacting variables. Two mainstream approaches regarding BN structure learning are the constraint-based and the score-based methods. However, due to their inherent limitations, in many cases the two approaches can only return partially directed graphs rather than DAGs. Please refer to [35] and [36] for details. A comparative evaluation of correlation networks, GGMs and BNs has been made in the reconstruction of gene regulation networks [37]. The results indicated that GGMs performed comparably to BNs on general observations, and both GGMs and BNs outperformed correlation networks on Gaussian observations.

Besides the construction of undirected or partially directed phenotype networks, QTL mapping for the traits is also a prerequisite for using the QPSO algorithm. Standard QTL mapping methods, including association mapping and linkage mapping, process phenotypic traits in a parallel fashion without paying attention to the underlying dependence structure of traits. Neto *et al*. [11] claimed that QTL mapping conditional on the phenotype network should lead to a better estimated genetic architecture, and a better genetic architecture should in turn result in a better inferred phenotype network. Accordingly, they developed a statistical framework, named QTLnet, to jointly infer a causal phenotype network and the associated genetic architecture for a set of correlated phenotypes. The QTLnet method is actually a Metropolis–Hastings algorithm that integrates QTL mapping and the sampling of directed phenotype networks at each step. However, like many other Markov Chain Monte Carlo approaches, this method shows slow mixing of the resulting Markov chains and requires considerable computation time. Its implementation in R can handle no more than 20 traits at this point [11].

The QPSO algorithm treats QTL mapping independent from phenotype network reconstruction and cannot correct misspecified edges in undirected phenotype networks pre-learnt by the PC algorithm. In this sense, it would be considered less robust than the QTLnet method. We observed, however, that the QPSO algorithm performed well in the reconstruction of directed phenotype networks: 1) the results of our first set of simulations and also the ones shown in [10] implied that given relatively sufficient samples (say, ≥100 for a network composed of 34 phenotypes and 27 edges, or, ≥300 for a network composed of 100 phenotypes and 107 edges), the undirected phenotype networks recovered by the PC-skeleton algorithm were fairly reliable (with recall >0.85 and precision >0.90); 2) the simulation results presented in [12] indicated that in small-scale phenotype networks (to which the QTLnet method is only applicable), the QTLnet method was outperformed by the QDG algorithm that was used as benchmark in this study; 3) the results of our second and third sets of simulations showed that compared with the benchmark QDG algorithm, our proposed method was applicable to more general cases and led to more accurate overall orientations. In summary, we have confidence that the QPSO algorithm is of great potential in practical applications.

In simulation experiments, the QPSO algorithm was applied to a random network consisting of dozens of nodes and edges. Theoretically, this method has no limit to the scale of either random networks or scale-free networks, since it always decomposes a whole network into a finite number of LGPNs and makes causal inferences in the LGPNs using a heuristic search strategy. Scale-free networks show power-law degree distributions that are very different from the Poisson degree distributions of random networks. More specifically, in scale-free networks, most nodes have relatively few links while only a few nodes (called hubs) have a large number of links; contrariwise, in random networks nodes are more evenly connected. Here we would like to point out that node degree distribution is believed to have some effect on the efficiency of the QPSO algorithm, but the extent of this impact is hard to evaluate. Recall from the Method section that the LODG is selected from 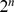 candidates, where *n* would be a big number if either or both of *Y*_1_ and *Y*_2_ are hubs. A large *n* means that, on the one hand, an enormous computational effort has to be made when scanning for the LODG; on the other hand, the number of LGPNs decomposed from the whole network is significantly reduced as a great number of undirected edges are assigned to the same LGPN. These two effects will counterbalance each other to some extent; but on the whole, the overall efficiency of the algorithm will vary a lot depending on the specific circumstances, including sample size, the number of nodes, and the node degree distribution. In addition, computer memory and processor speed are practical factors that can also affect the scalability and efficiency of the algorithm.

As explained previously, the QPSO algorithm returns fully or partially directed phenotype networks depending on the number of available QTLs. Its exhaustive search for LODGs is based on the distinction between non-equivalent DAGs, each of which has a unique set of v-structures. Thus, there is no directed cycle in a LODG. However, the QPSO algorithm is overall a heuristic method. It takes a random walk from one LODG to another. The integration of all LODGs does not necessarily lead to a complete DAG. That is, in some cases, it is possible that certain edges in two or more LODGs form a directed cycle. Please note that the benchmark QDG algorithm has substantially the same property.

We developed our methodology in the first place for data from plant breeding experiments, in which advanced experimental designs are common that include local control of error variation at multiple levels and in multiple directions. As genotypes for population types like doubled haploids and recombinant inbred lines are replicated in such experiments, reconstruction of networks take place at genotypic means obtained from mixed model analyses of one or more experiments. These genotypic means will have small standard errors and that will contribute to the stability of reconstructed directed networks. For metabolic assessments, usually pooled samples of fruits stemming from multiple replicates in an experiment are used. Pooling is another way of reducing measurement error. Therefore, it will beneficial to bring phenotypic traits to the aggregation level of genotypic means before trying to reconstruct a phenotype network. The QPSO algorithm is applicable to a complete data matrix of genotypes (samples) by traits. Pre-processing of phenotypic data by converting them to genotypic means by mixed model analyses provides a straightforward and accurate way of imputing missing phenotypic values.

In conclusion, we have presented a novel heuristic search algorithm, named QPSO, to infer causal relationships between correlated traits. This algorithm allows some traits to come without QTLs, and it takes into account associated phenotypic interactions in addition to QTLs when orienting undirected edges between traits. Thanks to these two properties, the QPSO algorithm has much broader applicability and produces more accurate overall orientations, compared to the benchmark QDG algorithm.

## Supporting Information

File S1 **Detailed explanation of each candidate directed graph of a LGPN possessing a distinct set of v-structures.**
(DOCX)

File S2 **This sheet represents one of the 20 datasets used in simulation experiments with respect to sample size of 500.** Each row corresponds to an individual. The first 34 columns correspond to phenotypic traits and the other 31 columns correspond to markers coinciding with QTLs. In particular, the last 16 columns data were not used in the third set of simulations.
(XLSX)

File S3 **This sheet contains metabolic and QTL data collected in ripe tomato fruits.** Columns stand for metabolites and markers close or in QTLs; rows represent a total of 93 tomato cultivars, including 20 beef tomatoes, 17 cherry tomatoes and 56 round tomatoes.
(XLSX)

## References

[1] Jiang CJ, Zeng ZB (1995) Multiple-Trait Analysis of Genetic-Mapping for Quantitative Trait Loci. Genetics 140: 1111–1127. 7672582 10.1093/genetics/140.3.1111 PMC1206666

[2] Calus MPL, Veerkamp RF (2011) Accuracy of multi-trait genomic selection using different methods. Genetics Selection Evolution 43. 10.1186/1297-9686-43-26 PMC3146811 21729282

[3] Rosa GJM, Valente BD, de los Campos G, Wu XL, Gianola D, et al.. (2011) Inferring causal phenotype networks using structural equation models. Genetics Selection Evolution 43. 10.1186/1297-9686-43-6 PMC3056759 21310061

[4] Valente BD, Rosa GJM, Gianola D, Wu XL, Weigel K (2013) Is Structural Equation Modeling Advantageous for the Genetic Improvement of Multiple Traits? Genetics 194: 561–572. 23608193 10.1534/genetics.113.151209 PMC3697964

[5] Jansen RC, Tesson BM, Fu JY, Yang YJ, McIntyre LM (2009) Defining gene and QTL networks. Current Opinion in Plant Biology 12: 241–246. 19196544 10.1016/j.pbi.2009.01.003

[6] Schadt EE, Lamb J, Yang X, Zhu J, Edwards S, et al (2005) An integrative genomics approach to infer causal associations between gene expression and disease. Nature Genetics 37: 710–717. 15965475 10.1038/ng1589 PMC2841396

[7] Li Y, Tesson BM, Churchill GA, Jansen RC (2010) Critical reasoning on causal inference in genome-wide linkage and association studies. Trends in Genetics 26: 493–498. 20951462 10.1016/j.tig.2010.09.002 PMC2991400

[8] Aten JE, Fuller TF, Lusis AJ, Horvath S (2008) Using genetic markers to orient the edges in quantitative trait networks: The NEO software. Bmc Systems Biology 2. 10.1186/1752-0509-2-34 PMC2387136 18412962

[9] Li RH, Tsaih SW, Shockley K, Stylianou IM, Wergedal J, et al (2006) Structural model analysis of multiple quantitative traits. Plos Genetics 2: 1046–1057. 10.1371/journal.pgen.0020114 PMC1513264 16848643

[10] Neto EC, Ferrara CT, Attie AD, Yandell BS (2008) Inferring causal phenotype networks from segregating populations. Genetics 179: 1089–1100. 18505877 10.1534/genetics.107.085167 PMC2429862

[11] Neto EC, Keller MP, Attie AD, Yandell BS (2010) Causal Graphical Models in Systems Genetics: A Unified Framework for Joint Inference of Causal Network and Genetic Architecture for Correlated Phenotypes. Annals of Applied Statistics 4: 320–339. 21218138 10.1214/09-aoas288 PMC3017382

[12] Logsdon BA, Mezey J (2010) Gene Expression Network Reconstruction by Convex Feature Selection when Incorporating Genetic Perturbations. Plos Computational Biology 6. 10.1371/journal.pcbi.1001014 PMC2996324 21152011

[13] Spirtes P, Glymour CN, Scheines R (2000) Causation, prediction, and search. Cambridge, Mass.: MIT Press. xxi, 543 p.

[14] Gagneur J, Elze MC, Tresch A (2011) Selective Phenotyping, Entropy Reduction, and the Mastermind game. Bmc Bioinformatics 12. 10.1186/1471-2105-12-406 PMC3258278 22014271

[15] Hill CB, Taylor JD, Edwards J, Mather D, Bacic A, et al (2013) Whole-Genome Mapping of Agronomic and Metabolic Traits to Identify Novel Quantitative Trait Loci in Bread Wheat Grown in a Water-Limited Environment. Plant Physiology 162: 1266–1281. 23660834 10.1104/pp.113.217851 PMC3707548

[16] Joosen RVL, Arends D, Li Y, Willems LAJ, Keurentjes JJB, et al (2013) Identifying Genotype-by-Environment Interactions in the Metabolism of Germinating Arabidopsis Seeds Using Generalized Genetical Genomics. Plant Physiology 162: 553–566. 23606598 10.1104/pp.113.216176 PMC3668052

[17] Malosetti M, Ribaut JM, Vargas M, Crossa J, van Eeuwijk FA (2008) A multi-trait multi-environment QTL mixed model with an application to drought and nitrogen stress trials in maize (Zea mays L.). Euphytica 161: 241–257.

[18] Alimi NA, Bink MCAM, Dieleman JA, Magan JJ, Wubs AM, et al (2013) Multi-trait and multi-environment QTL analyses of yield and a set of physiological traits in pepper. Theoretical and Applied Genetics 126: 2597–2625. 23903631 10.1007/s00122-013-2160-3

[19] Verma T, Pearl J (1990) Equivalence and synthesis of causal models. 220–227.

[20] Shenoy PP (2006) Inference in hybrid Bayesian networks using mixtures of Gaussians. 428–436.

[21] Schwarz G (1978) Estimating the dimension of a model. Annals of Statistics 6: 461–464.

[22] Roessner-Tunali U, Hegemann B, Lytovchenko A, Carrari F, Bruedigam C, et al (2003) Metabolic profiling of transgenic tomato plants overexpressing hexokinase reveals that the influence of hexose phosphorylation diminishes during fruit development. Plant Physiology 133: 84–99. 12970477 10.1104/pp.103.023572 PMC196583

[23] Tikunov Y, Lommen A, de Vos CHR, Verhoeven HA, Bino RJ, et al (2005) A novel approach for nontargeted data analysis for metabolomics. Large-scale profiling of tomato fruit volatiles. Plant Physiology 139: 1125–1137. 16286451 10.1104/pp.105.068130 PMC1283752

[24] Ursem R, Tikunov Y, Bovy A, van Berloo R, van Eeuwijk F (2008) A correlation network approach to metabolic data analysis for tomato fruits. Euphytica 161: 181–193.

[25] Blair RH, Kliebenstein DJ, Churchill GA (2012) What Can Causal Networks Tell Us about Metabolic Pathways? Plos Computational Biology 8. 10.1371/journal.pcbi.1002458 PMC3320578 22496633

[26] Baldermann S, Kato M, Kurosawa M, Kurobayashi Y, Fujita A, et al (2010) Functional characterization of a carotenoid cleavage dioxygenase 1 and its relation to the carotenoid accumulation and volatile emission during the floral development of Osmanthus fragrans Lour. Journal of Experimental Botany 61: 2967–2977. 20478967 10.1093/jxb/erq123

[27] Gao HY, Zhu HL, Shao Y, Chen AJ, Lu CW, et al (2008) Lycopene accumulation affects the biosynthesis of some carotenoid-related volatiles independent of ethylene in tomato. Journal of Integrative Plant Biology 50: 991–996. 18713349 10.1111/j.1744-7909.2008.00685.x

[28] Rambla JL, Tikunov YM, Monforte1 AJ, Bovy AG, Granell A (2014) The expanded tomato fruit volatile landscape. Journal of Experimental Botany. 10.1093/jxb/eru128 24692651

[29] Mathieu S, Cin VD, Fei ZJ, Li H, Bliss P, et al (2009) Flavour compounds in tomato fruits: identification of loci and potential pathways affecting volatile composition. Journal of Experimental Botany 60: 325–337. 19088332 10.1093/jxb/ern294 PMC3071775

[30] Krumsiek J, Suhre K, Illig T, Adamski J, Theis FJ (2011) Gaussian graphical modeling reconstructs pathway reactions from high-throughput metabolomics data. Bmc Systems Biology 5. 10.1186/1752-0509-5-21 PMC3224437 21281499

[31] Ma SS, Gong QQ, Bohnert HJ (2007) An Arabidopsis gene network based on the graphical Gaussian model. Genome Research 17: 1614–1625. 17921353 10.1101/gr.6911207 PMC2045144

[32] Gavai AK, Tikunov Y, Ursem R, Bovy A, van Eeuwijk F, et al (2009) Constraint-based probabilistic learning of metabolic pathways from tomato volatiles. Metabolomics 5: 419–428. 20046866 10.1007/s11306-009-0166-2 PMC2794349

[33] Hodges AP, Dai DJ, Xiang ZS, Woolf P, Xi CW, et al.. (2010) Bayesian Network Expansion Identifies New ROS and Biofilm Regulators. Plos One 5. 10.1371/journal.pone.0009513 PMC2831072 20209085

[34] Iyer SP, Shafran I, Grayson D, Gates K, Nigg GT, et al (2013) Inferring functional connectivity in MRI using Bayesian network structure learning with a modified PC algorithm. NeuroImage 75: 165–175. 23501054 10.1016/j.neuroimage.2013.02.054 PMC3683082

[35] Mahdi R, Mezey J (2013) Sub-Local Constraint-Based Learning of Bayesian Networks Using A Joint Dependence Criterion. Journal of Machine Learning Research 14: 1563–1603.

[36] Chickering DM (1996) Learning equivalence classes of Bayesian network structures. Uncertainty in Artificial Intelligence: 150–157.

[37] Werhli AV, Grzegorczyk M, Husmeier D (2006) Comparative evaluation of reverse engineering gene regulatory networks with relevance networks, graphical gaussian models and bayesian networks. Bioinformatics 22: 2523–2531. 16844710 10.1093/bioinformatics/btl391

