# Supplementary material for: A New Method to Infer Causal Phenotype Networks Using QTL and Phenotypic Information
Source: PLoS One. 2014 Aug 21;9(8):e103997. doi: 10.1371/journal.pone.0103997 (PMC4140682; doi:10.1371/journal.pone.0103997)
Supplement: File S1 — Detailed explanation of each candidate directed graph of a LGPN possessing a distinct set of v-structures. (DOCX) [file pone.0103997.s001.docx]

Figure 2A shows a local generalized phenotype network (LGPN), in which 1) both traits *Y*_1_ and *Y*_2_ have parent nodes and at least one of *Y*_1_ and *Y*_2_ has unique parent nodes; 2) each neighboring trait of *Y*_1_ is nonadjacent to at least one of the parent nodes of *Y*_1_, and the same is true of *Y*_1_.

***Theorem*** [Verma and Pearl, 1990]*: Two directed acyclic graphs (DAGs) are likelihood equivalent if and only if they have the same skeletons and the same v-structures (A v-structure in a DAG G is an ordered triple of nodes (X, Y, Z) such that G contains the directed edges X→Y and Z→Y, and X and Z are not adjacent in G).*

According to this theorem, we can deduce that given the directed edge pointing from *P*_11_ to *Y*_1_, the two candidate directions of the undirected edge between *C*_11_ and *Y*_1_ (i.e., *C*_11_*→Y*_1_ and *Y*_1_*→C*_11_) will form two nonequivalent structures: *P*_11_*→Y*_1_*←C*_11_ and *P*_11_*→Y*_1_*→C*_11_. The reason is quite straightforward: since *C*_11_ is nonadjacent to *P*_11_, *P*_11_*→Y*_1_*←C*_11_ is a v-structure whereas *P*_11_*→Y*_1_*→C*_11_ is not. The same is true if *P*_11_ is replaced by any other node $\in${*P*_11_,…,*P*_1k_}$\cup${*P*_1_,…,*P_s_*}, and, *C*_11_ is replaced by any other node $\in${*C*_11_,…,*C*_1_*_u_*}$\cup${*C*_1_,…,*C_t_*}.

Similarly, we can deduce that given the directed edge pointing from *P*_21_ to *Y*_2_, *P*_21_*→Y*_2_*←C*_21_ and *P*_21_*→Y*_2_*→C*_21_ are nonequivalent. And the same is true if *P*_21_ is replaced by any other node $\in${*P*_21_,…,*P*_2_*_l_*}$\cup${*P*_1_,…,*P_s_*}, and, *C*_21_ is replaced by any other node $\in${*C*_21_,…,*C*_2_*_v_*}$\cup${*C*_1_,…,*C_t_*}.

Lastly, let’s consider the orientation of the undirected edge between *Y*_1_ and *Y*_2_. We restrict ourselves to the cases where at least one of *Y*_1_ and *Y*_2_ has unique parent nodes (please note the unique parent nodes are not limited to QTLs, that is, traits that have been previously determined as parent nodes of *Y*_1_ and *Y*_2_ are also taken into account). In Figure 2A, *P*_11_ is a unique parent node of *Y*_1_, which indicates that *P*_11_ is nonadjacent to *Y*_2_. Therefore, we know that *P*_11_*→Y*_1_*←Y*_2_ and *P*_11_*→Y*_1_*→Y*_2_ are nonequivalent as the former forms a v-structure while the latter does not. The same is true if *P*_11_ is replaced by any other node $\in${*P*_11_,…,*P*_1k_}.

Similarly, we have *P*_21_*→Y*_2_*←Y*_1_ and *P*_21_*→Y*_2_*→Y*_1_ are nonequivalent and the same is true if *P*_21_ is replaced by any other node $\in${*P*_21_,…,*P*_2_*_l_*}.

In conclusion, if a LGPN satisfies the aforementioned two conditions and there are a total of *n* undirected edges involved in it, we know that each of the 2*^n^* candidate directed graphs possesses a distinct set of v-structures and is therefore not equivalent to the others.
